# Positive Bacterial Culture among Suspected Orthopedic Infections in a Tertiary Care Centre: A Descriptive Cross-sectional Study

**DOI:** 10.31729/jnma.7620

**Published:** 2022-08-31

**Authors:** Ishor Pradhan, Subhash Regmi, Meena Kunwar, Bibek Basukala, Amit Joshi

**Affiliations:** 1 Department of Orthopedics, B & B Hospital, Gwarko, Lalitpur, Nepal; 2 Department of Ophthalmology, B & B Hospital, Gwarko, Lalitpur, Nepal

**Keywords:** *culture techniques*, *infections*, *microbial sensitivity tests*, *prevalence*

## Abstract

**Introduction:**

A hospital-based investigation of bacteriological isolates helps to identify common causative bacteria and their antibiotic sensitivity patterns. This helps in formulating presumptive antibiotic therapy and in reducing antibiotic misuse. The aim of this study is to find out the prevalence of positive bacterial culture isolates among suspected orthopaedic infections in a tertiary care centre.

**Methods:**

A descriptive cross-sectional study was conducted from the electronic data record of the Department of Microbiology of a tertiary care centre from 1 January 2017 to 31 December 2021. The study was conducted following ethical approval from the Institutional Review Committee (Reference number: IRC-2021-11-09-1). Culture reports of suspected orthopaedic infections were evaluated, and those with missing data were excluded. A convenience sampling method was used. Point estimate and 95% Confidence Interval were calculated.

**Results:**

Out of 6201 specimens, positive bacterial culture were found in 2957 (47.69%) (46.4548.93, 95% Confidence Interval). Among them, 1561 (56.01%) were gram-negative organisms and 677 (24.29%) were gram-positive. A total of 2787 (94.25%) were wound/pus swab cultures and 170 (5.74%) were tissue cultures.

**Conclusions:**

The prevalence of positive bacterial culture among suspected orthopaedic infections was lower than in other international studies. Among bacteriological isolates, gram-negative organisms are more than gram-positive organisms.

## INTRODUCTION

Infection is one of the common complications in orthopaedic trauma patients, which accounts for around 5-10%.^1^ Treatment includes "empirical antibiotic therapy" until the availability of culture results, followed by culture-sensitive antibiotics.^2^ However, empirical therapy may lead to the overuse of a particular set of antibiotics and contribute heavily to the development of resistance.^3^ This possess a significant therapeutic challenge, especially in low-income countries where there is limited availability of antibiotics. Recently, the concept of "presumptive antibiotic therapy" has emerged as a potential solution to antibiotic misuse, which involves prescribing the most sensitive antibiotics to the most common pathogen.^4^

It is known that a hospital-based investigation of bacteriological isolates is effective in the identification of common local pathogens and their antibiotic sensitivity patterns.^5^ It helps in enhancing presumptive antibiotic therapy and reduces antibiotic misuse, thereby preventing the development of antibiotic resistance.^6^

The objective of this study was to find out the prevalence of positive bacterial culture among suspected orthopaedic infections in a tertiary care centre.

## METHODS

A descriptive cross-sectional study was conducted in the Department of Microbiology, B & B Hospital, Gwarko, Lalitpur, Nepal following the ethical approval from the Institutional Review Committee of the same institute (Reference number: IRC-2021-11-09-1). Electronic data record from 1 January 2017 to 31 December 2021 was retrived. Culture reports of samples obtained from patients with suspected orthopaedic infections were included. Among them reports with missing data were excluded. Convenience sampling method was used.

The sample size was calculated using the formula:


$$\begin{array}{ll} \text{n} & ={\text{Z}}^{2}\times \frac{\text{p}\times \text{q}}{{\text{e}}^{\text{2}}}\\ & =1.96^{2}\times \frac{0.50\times 0.50}{0.02^{2}}\\ & =2401 \end{array}$$

Where,

n= minimum required sample size
Z= 1.96 at 95% Confidence Interval (CI)
p= prevalence taken as 50% for maximum sample size calculation
q= 1-p
e= margin of error, 2%

The minimum required calculated sample size was 2401. After adjustment for 10% missing data and doubling, the sample size calculated was 5336. However, a total of 6,201 culture reports were included in the study.

Wound/pus swabs or tissue specimens were sent for cultures in patients with infective conditions, such as surgical site infection, periprosthetic infection, osteomyelitis, and septic arthritis.^2^ Suspicion of infection was based on: clinical features, such as wound dehiscence, sinus discharge, significant wound site pain, and fever; imaging findings, such as a collection of fluids and periosteal reaction; and laboratory parameters, such as elevated white blood cell count, erythrocyte sedimentation rate and C-reactive protein levels.^7^

The culture samples were obtained in a sterile swab-stick tube or plastic container depending upon the specimen. Specimens were aseptically inoculated on blood agar (with 5% sheep blood) plates and incubated aerobically at 37°C for 48 hours. Identification of pathogens was done based on microscopic characteristics. Antibiotic sensitivity testing was done following standard recommendations provided by the Clinical and Laboratory Standards Institute.^8^ Inoculate were prepared for each bacterial isolate by adjusting the turbidity to 0.5 McFarland standard and spread on Muller-Hinton agar plates. Antibiotic discs containing amikacin (30 mcg), ceftriaxone (30 mcg), cefoperazone+sulbactam (75 mcg/30 mcg), clindamycin (2 mcg), colistin sulphate (10 mcg), gentamicin (10 mcg), linezolid (30 mcg), meropenem (10 mcg), ofloxacin (5 mcg), piperacillin+tazobactam (100 mcg/10 μg) were obtained from Microexpress, Division of Tulip Diagnostic (P) Limited (Goa, India).

Positive bacterial culture was categorised after they have Isolated pathogens were classified mainly into 3 groups: gram-positive, gram-negative, and mixed. Antibiotic sensitivity outcomes were categorized as sensitive and resistant.

The following data were extracted: Age, Sex, type of specimen (wound/pus swab, and tissue), culture outcomes, and antibiotic sensitivity patterns. Data were entered and analysed in IBM SPSS Statistics 24.0. Point estimate and 95% CI were calculated.

## RESULTS

**Figure 1:**
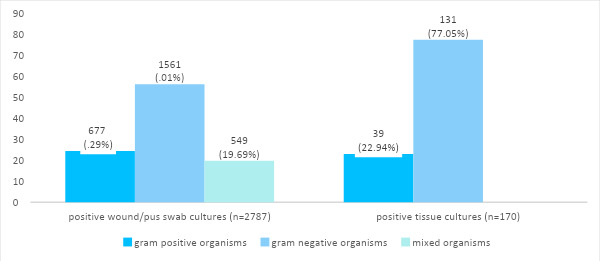
Characteristics of bacteriological isolates based on gram stain (n= 2787).

Out of 6201 samples, positive bacterial culture were found in 2957 (47.69%) (46.45-48.93, 95% CI). Among them 2002 (67.70%) were males and 955 (32.29%) were females, and the mean age of the patients was 41.67±22.03 years. A total of 2787 (94.25%) were wound/pus swab cultures and 170 (5.74%) were tissue cultures (Figure 1).

*S. aureus* was found in 678 (24.32%) and 39 (22.94%) among positive wound/pus swab culture and positive tissue culture respectively (Table 1).

**Table 1:** Characteristics of bacteriological isolates based on the types of bacteria.

Types of Bacteria	Positive wound/pus swab cultures (n= 2,787) n (%)	Positive tissue cultures (n= 170) n (%)
*S. aureus*	678 (24.32)	39 (22.94)
MSSA^*^	219 (7.85)	16 (9.41)
MRSA^†^	283 (10.15)	16 (9.41)
CoNSA^‡^	176 (6.31)	7 (4.11)
*E. coli*	493 (17.68)	36 (21.17)
*Klebsiella*	384 (13.77)	33 (19.41)
*Pseudomonas*	383 (13.74)	19 (11.17)
*Acinetobacter*	301 (10.8)	21 (12.35)
Multiple bacteria	81 (2.9)	-
Others	294 (10.54)	22 (12.94)

* MSSA= methicillin-sensitive staphylococcus aureus

† MRSA-methicillin-resistant staphylococcus aureus

‡ CoNSA-coagulase-negative staphylococcus aureus

Amikacin was sensitive in 95 (81.89%) of the MSSA isolates whereas 80 (30.65%) to Acinietobacter (Table 2).

**Table 2:** The outcomes of antibiotics sensitivity testing of bacteriological isolates (n = 2787).

Antibiotics	Organisms													
	E. coli		MSSA		MRSA		CoNSA		*Klebsiella*		Pseudo monas		Acinieto bacter	
	^*^N	^†^S n (%)	N	S n (%)	N	S n (%)	N	S n (%)	N	S n (%)	N	S n (%)	N	S n (%)
Amikacin	443	336 (75.84)	116	95 (81.89)	118	91 (77.11)	72	43 (59.72)	338	154 (45.56)	330	243 (73.63)	261	80 (30.65)
Ceftriaxone	402	125 (31.09)	27	8 (29.62)	27	6 (22.22)	24	4 (16.66)	305	53 (17.37)	84	15 (17.85)	237	26 (10.97)
Cefoperazone + sulbactam	368	264 (71.73)	32	22 (68.75)	34	21 (61.76)	27	19 (70.37)	294	127 (43.19)	301	197 (65.44)	245	72 (29.38)
Gentamycin	428	254 (59.34)	130	101 (77.69)	178	119 (66.85)	108	74 (68.51)	317	124 (39.11)	422	203 (48.10)	255	76 (29.8)
Clindamycin	88	56 (63.63)	124	90 (72.58)	145	63 (43.44)	78	36 (46.15)	64	23 (35.93)	17	8 (47.05)	20	9 (45)
Ofloxacin	418	186 (44.49	180	94 (52.22)	244	84 (34.42)	137	80 (58.39)	326	121 (37.11)	391	149 (38.1)	109	31 (28.44)
Linezolid	66	65 (98.48)	208	208 (100)	228	227 (99.56)	147	146 (99.31)	43	42 (97.67)	5	5 (100)	8	6 (75)
Piperacillin + tazobactam	351	190 (54.13)	107	83 (77.57)	162	91 (56.17)	88	60 (68.18)	289	98 (33.91)	288	177 (61.45)	191	61 (31.93)
Meropenem	245	164 (66.93)	48	33 (68.75)	91	70 (76.92)	52	44 (84.61)	157	79 (50.31)	234	142 (60.68)	180	75 (41.66)
Colistin sulphate	208	208 (100)	29	29 (100)	70	68 (97.14)	38	38 (100)	205	204 (99.51)	268	268 (100)	207	207 (100)

* N = number of tested culture isolates

† S = antibiotics sensitivity

## DISCUSSION

This study identified that the prevalence of bacteriological isolates in orthopedic infection was 44.03%. The observed positivity rate was significantly lower compared to what was reported in the literature, which was around 75-93%.^9,10^ Several factors may have contributed to the lower positivity rate, such as quality of sampling and culture methods, delay in planting, short incubation period, prior usage of antibiotics, and infection due to anaerobic microorganisms.^11,12^

The culture positivity rate was observed more in wound/pus swab cultures than in tissue cultures. Similar outcomes were reported in previous studies conducted in the United States and Greece.^13,14^ A study conducted in the United States found that the swab culture resulted in an 11% higher microbial recovery rate compared to tissue cultures.^13^ Similarly, another study conducted in Greece found that swab cultures have significantly higher sensitivity and negative predictive values compared to tissue cultures.^14^ However, these studies were conducted in chronic non-healing and low-grade diabetic ulcer wounds. In contrast, studies conducted in China and Italy found tissue culture more effective in isolating causative pathogens compared to swab cultures in high-grade diabetic foot wounds and advanced-stage pressure sore wounds, respectively.^15,16^ In addition, a systematic review found tissue cultures and biopsies superior to swab cultures in detecting infection, especially for deep wound infections.^17^ However, the included studies were moderate-to low-quality studies and had significant variation in sampling techniques, wound types, and participants' baseline characteristics. This suggests that there is still a controversy regarding the choice of sampling technique for microbiological cultures, especially in orthopaedic wounds. Hence, both swab and tissue culture techniques are recommended, and further prospective studies comparing the sensitivity and specificity of these two techniques can be conducted to evaluate the efficacy of one technique over the other.

Based on gram stain results, gram-negative organisms were isolated more than gram-positive organisms in both wound/pus swabs and tissue cultures. These findings were similar to that reported by studies conducted in Brazil and Ethiopia.^9,10^ However, the most common organism isolated was S. aureus in both wound/pus swab cultures and tissue cultures. A study conducted in Brazil evaluating 147 orthopaedic infections found 93.2% positive culture results; out of which, 56.5% were gram-negative isolates, and the most common organism isolated was *S. aureus.^9^* This suggests that *S. aureus* remains the most common organism responsible for the orthopaedic infection.

Antibiotic sensitivity testing demonstrated that most isolated pathogens were mostly resistant to ceftriaxone. High resistance (60-80%) to third-generation cephalosporins has also been observed in some previous studies.^9,10^ Higher resistance to third-generation cephalosporins could be due to their frequent usage as first-line antibiotic therapy. In contrast, most isolated pathogens were sensitive to linezolid (75-100%) and colistin sulphate (97.14-100%). The sensitivity of these antibiotics was high because these are relatively newer antibiotics and are often used as second-line therapy.

Considering individual pathogens, MRSA, which is a most frequently isolated *S. aureus,* is found to be mostly resistant to ceftriaxone 21 (77.78%), ofloxacin 160 (65.58%), and clindamycin 82 (56.56%), and sensitive to linezolid 227 (99.56%), colistin 68 (97.14%), amikacin 91 (77.11%), meropenem 70 (76.92%), gentamycin 119 (66.85%), cefoperazone+sulbactam 21 (61.76%) and piperacillin+tazobactam 91 (56.17%). Similarly, *E. coli,* a second most common culture isolate, is found to be mostly resistant to ceftriaxone 277 (68.91%) and ofloxacin 232 (55.51%), and sensitive to colistin 208 (100%), linezolid 65 (98.48%), amikacin 336 (75.84%), cefoperazone+sulbactam 264 (71.73%), meropenem 164 (66.93%), clindamycin 56 (63.63%), gentamycin 254 (59.34%), and piperacillin+tazobactam 190 (54.13%). This suggests that ceftriaxone and ofloxacin are not effective first-line therapy in treating patients with orthopaedic infections. Amikacin or gentamycin could be used as effective first-line therapy and Colistin, linezolid, cefoperazone+sulbactam, or meropenem could be reserved for second-line therapy. However, due to the potential nephrotoxicity of amikacin or gentamycin, frequent monitoring of kidney function and dose adjustments accordingly is advised.^18^

This study has several limitations. It was a singlecentre study, and the infection's location, severity, and duration, from which the samples were taken for cultures, were not evaluated. There was considerable variation in the number of organisms isolated and the number of organisms tested for antibiotic susceptibility. Some of the commonly used first-line antibiotics, such as cloxacillin, flucloxacillin, and amoxicillin+clavulanic were not tested. This suggests that the outcomes of this study cannot be generalized to all patients with orthopaedic infections. However, the study evaluated a large number of culture samples over the duration of 4 years. This provides substantial evidence regarding common local pathogens and their susceptibility patterns, which certainly helps in enhancing presumptive antibiotic therapy while managing orthopaedic infections at this hospital.

## CONCLUSIONS

The prevalence of positive bacterial culture among suspected orthopaedic infections was lower as compared to other international studies. The number of gram-negative organisms was higher than grampositive organisms. *S. aureus* is the most common organism isolated followed by *E. coli, Klebsiella, and Pseudomonas.* A multi-centric antibiotic sensitivity testing is recommended to establish a recommendation for presumptive antibiotic therapy in treating patients with suspected orthopaedic infections.

## References

[1] Wang B, Xiao X, Zhang J, Han W, Hersi SA, Tang X (2021). Epidemiology and microbiology of fracture-related infection: a multicenter study in Northeast China.. J Orthop Surg Res..

[2] Purghel F, Badea R, Ciuvica R, Anastasiu A (2006). The use of antibiotics in traumatology and orthopaedic surgery.. Mffidica-a J Clin Med..

[3] Li B, Webster TJ (2018). Bacteria antibiotic resistance: New challenges and opportunities for implant-associated orthopedic infections.. J Orthop Res..

[4] Hopkins TL, Daley MJ, Rose DT, Jaso TC, Brown CVR (2016). Presumptive antibiotic therapy for civilian trauma injuries.. J Trauma Acute Care Surg..

[5] Hostler CJ, Moehring RW, Ashley ESD, Johnson M, Davis A, Lewis SS (2018). Feasibility and value of developing a regional antibiogram for community hospitals.. Infect Control Hosp Epidemiol..

[6] Anderson DJ, Miller B, Marfatia R, Drew R (2012). Ability of an antibiogram to predict Pseudomonas aeruginosa susceptibility to targeted antimicrobials based on hospital day of isolation.. Infect Control Hosp Epidemiol..

[7] Zhang J, Li X, Huang G, Wang A, Zhang F (2021). Clinical features and etiology of musculoskeletal infection with or without sepsis in the emergency department.. Int J Gen Med..

[8] Weinstein MP, Lewis JS (2020). The clinical and laboratory standards institute subcommittee on antimicrobial susceptibility testing: background, organization, functions, and processes.. J Clin Microbiol..

[9] Tuon FF, Cieslinski J, Ono AFM, Goto FL, Machinski JM, Mantovani LK (2019). Microbiological profile and susceptibility pattern of surgical site infections related to orthopaedic trauma.. Int Orthop..

[10] Mengesha RE, Kasa BG-S, Saravanan M, Berhe DF, Wasihun AG (2014). Aerobic bacteria in post surgical wound infections and pattern of their antimicrobial susceptibility in Ayder Teaching and Referral Hospital, Mekelle, Ethiopia.. BMC Res Notes..

[11] Jefferson H, Dalton HP, Escobar MR, Allison MJ (1975). Transportation delay and the microbiological quality of clinical specimens.. Am J Clin Pathol [Internet]..

[12] Marjan W-B, Natividad B, Alex S, Robin P (2017). The effect of preoperative antimicrobial prophylaxis on intraoperative culture results in patients with a suspected or confirmed prosthetic joint infection: a systematic review.. J Clin Microbiol [Internet]..

[13] Smith ME, Robinowitz N, Chaulk P, Johnson K (2014). Comparison of chronic wound culture techniques: swab versus curetted tissue for microbial recovery.. Br J Community Nurs..

[14] Demetriou M, Papanas N, Panopoulou M, Papatheodorou K, Bounovas A, Maltezos E (2013). Tissue and swab culture in diabetic foot infections: neuropathic versus neuroischemic ulcers.. Int J Low Extrem Wounds..

[15] Huang Y, Cao Y, Zou M, Luo X, Jiang Y, Xue Y (2016). A comparison of tissue versus swab culturing of infected diabetic foot wounds.. Int J Endocrinol..

[16] Tedeschi S, Negosanti L, Sgarzani R, Trapani F, Pignanelli S, Battilana M (2017). Superficial swab versus deep-tissue biopsy for the microbiological diagnosis of local infection in advanced-stage pressure ulcers of spinal-cord-injured patients: a prospective study.. Clin Microbiol Infect..

[17] Copeland-Halperin LR, Kaminsky AJ, Bluefeld N, Miraliakbari R (2016). Sample procurement for cultures of infected wounds: a systematic review.. J Wound Care..

[18] Sweileh WM (2009). A prospective comparative study of gentamicin- and amikacin-induced nephrotoxicity in patients with normal baseline renal function.. Fundam Clin Pharmacol..

